# Depressive symptoms among frontline and non-frontline healthcare providers in response to the COVID-19 pandemic in Kelantan, Malaysia: A cross sectional study

**DOI:** 10.1371/journal.pone.0256932

**Published:** 2021-08-31

**Authors:** Mohd Noor Norhayati, Ruhana Che Yusof, Mohd Yacob Azman

**Affiliations:** 1 Department of Family Medicine, School of Medical Sciences, Universiti Sains Malaysia, Kubang Kerian, Kelantan, Malaysia; 2 Raja Perempuan Zainab II Hospital, Kota Bharu, Kelantan, Malaysia; China University of Mining and Technology, CHINA

## Abstract

**Background:**

Healthcare providers are vulnerable in the fight against COVID-19 and may experience significant psychological and mental health consequences. This study aimed to compare the levels of depressive symptoms among frontline and non-frontline healthcare providers in response to the COVID-19 pandemic.

**Methods:**

A comparative cross-sectional study was conducted in two government hospitals managing COVID-19-related cases in Kelantan, Malaysia from May to July 2020 to identify and compared depressive symptoms levels of frontline and non-frontline healthcare providers. Convenient sampling was applied in the selection of eligible participants and those diagnosed as having any psychiatric illnesses were excluded. The self-administered questionnaires for the Malay versions of the Hospital Anxiety and Depression Scale to measure depressive symptoms score and the Medical Outcome Study Social Support Survey to measure social support score as an important confounder. A descriptive analysis, independent *t*-test and ANCOVA were performed using SPSS version 26.

**Results:**

A total of 306 respondents from healthcare providers were recruited which 160 were frontline healthcare providers and 146 were non-frontline healthcare providers. The level of depressive symptoms (HADS score >8) was 27.5% for the frontline healthcare providers and 37.7% for the non-frontline healthcare providers. The mean depressive symptoms score for the non-frontline healthcare providers was 0.75 points higher than that of the frontline healthcare providers after adjusting for gender, duration of employment and social support.

**Conclusion:**

Non-frontline healthcare providers are also experiencing psychological distress during the COVID-19 pandemic even though they do not have direct contact with COVID-19 patients.

## Introduction

Although a year has passed since the first case of Corovavirus disease 2019 (COVID-19) was diagnosed, the case numbers are continuing to increase, with more than 50 million people globally having been infected and more than one million death [1]. Healthcare providers have a significant responsibility to combat the COVID-19 pandemic as they are on the front lines in managing public health. However, the increasing numbers of infected patients make them highly vulnerable to experiencing physical, mental and emotional exhaustion. These psychological burdens, especially among frontline staff, may have an impact on their mental health.

Many researchers have studied mental health problems due to COVID-19. Anxiety, depression, distress, sleep problems and somatic symptoms are among the common mental health problems experienced by healthcare providers during disease outbreaks [2]. These psychological impacts are significantly increased as healthcare providers have to work in high-risk environments and may have family members with COVID-19, suboptimal hand hygiene before and after contact with patients, inadequate personal protective equipment, close contact with patients (≥12 times/day), long working hours (≥15 h/day) and unprotected exposure to COVID 19 [3]. Staff redeployed as frontline healthcare providers also have increased levels of distress due to inadequate knowledge and experience managing COVID-19 patients and inadequate protective equipment, control measures and training to prevent the possibility of disease transmission [4].

A recent study reported the protective factors for mental health problems among healthcare providers [5]. These include self-coping styles such as active or passive psychological and life adjustments, taking the initiative to be altruistic, seeking team support, rational cognition through comparisons with other situations, favourable information and self-encouragement. A few interventions have been suggested, among them, supportive interventions provided by family members, government, society and colleagues; encouragement and motivational interventions (e.g. recognizing healthcare staff’s efforts and implementing effective measures to reduce the number of COVID-19 cases); protective interventions such as providing adequate and effective personal protective equipment and designing a safe place for rest breaks; educational and training interventions via online education for psychological and mental health; and the development and publishing of relevant guidelines [6]. These psychological interventions promote the emotional release and may help prevent serious mental health problems.

One parameter for mental health problems is depression. Depression has been reported in many studies of healthcare providers during the COVID-19 pandemic. The reported pooled prevalence of depression among healthcare providers in 11 studies from China, West Bengal, India and Singapore was 30.2% (95% confidence interval [CI]: 21.55; 39.78) [7], and 22.8% (95% CI: 15.10; 31.51) from 10 studies in China and Singapore [8]. In Malaysia, the reported mean level of depression among doctors was 3.99 (standard deviation [SD]: 4.69); the majority (69.0%) of them had normal levels of depression, 13.7% had mild depression, 7.2% had moderate depression, 6.0% had severe depression, and 4.0% had very severe depression [9]. The risk factors identified for depression in healthcare providers were increased workloads, respiratory and digestive symptoms, specific test(s) related to COVID-19, negative coping styles, and job burnout [10]. Being female, having a chronic disease, suspected of or having a confirmed diagnosis of COVID-19, and insufficient personal protective equipment have also been significantly associated with depression [11].

Working as a highly exposed group to COVID-19 infection, healthcare providers were at higher risk of psychological symptoms [12]. Whether directly or indirectly, this occupational and work-related factor with COVID-19 patients may also influence psychological impacts [13]. Currently, there are a very limited number of published studies involving the mental health of healthcare providers in Malaysia and notably few studies on depressive symptoms. Therefore, this study aimed to compare the levels of depressive symptoms between frontline and non-frontline healthcare providers in response to the COVID-19 pandemic in Kelantan.

## Methods

This study was a part of a large study on the psychological impact of the COVID-19 pandemic in Kelantan. The included psychological impacts were vicarious traumatization, anxiety and depressive symptoms. Each of the studies had shared the same methodology and respondents with the difference in the study’s objective. The studies of vicarious traumatization and anxiety (HADS-A subscale of the Hospital Anxiety and Depression Scale) were peer-reviewed and published in journals recently [14,15].

### Setting and participants

This comparative cross-sectional study was conducted among healthcare providers in two hospitals managing COVID-19-related cases from May to July 2020 in Kelantan, Malaysia. These government hospitals were designated as COVID-19 hospitals for managing COVID-19 patients throughout the state of Kelantan. Healthcare providers, such as medical specialists, medical officers, medical assistants, nurses, and health care assistants were included in the study. Medical specialists and medical officers were grouped into medical staffs; meanwhile, medical assistants, nurses, and healthcare assistants were grouped into paramedics. Those who had been diagnosed with any psychiatric illnesses were excluded. Frontline and non-frontline healthcare providers were identified and invited to participate in this study. Convenience sampling was applied in the selection of eligible participants [14,15].

### Sample size

The sample size was calculated by comparing two means using the Power and Sample Size Calculation software version 3.0.43 (DalePlummer, Tennessee). This study was part of a larger study on the psychological impact of the COVID-19 pandemic. The variable with the largest sample size was anxiety. In the previous study [16], the standard deviation of the anxiety score among non-frontline healthcare providers was 10.6, and a detectable difference of 3.5 was decided after considering its clinical importance. In this study, the calculated sample size was 160 for non-frontline healthcare providers and 160 for frontline healthcare providers after considering an alpha of 0.05, a power of 80% and a non-response rate of 10% [14,15].

### Data collection

An online method was used to invite eligible respondents. A Google Form was distributed through a WhatsApp group application. The respondents who agreed to participate in the research were requested to respond via a virtual consent form and to complete the self-administered questionnaires. They were informed that their participation in the research was voluntary and that they could withdraw their participation at any time. Sign-in to a Google account was not required to complete the survey. The participants were anonymous to reduce method and social desirability biases [14,15].

### Outcome measures

The case report form consisted of several parts, including a questionnaire on the healthcare providers’ socioeconomic data, the Malay version of the Hospital Anxiety and Depression Scale (HADS) questionnaire [17] and the Medical Outcome Study (MOS) Social Support Survey [18]. The sociodemographic data questionnaire required responses for age, race, marital status, number of children, education level, household income and occupational information, which included occupation, duration of employment, duration of work, shift work and type of work [15].

The Malay version of Hospital Anxiety and Depression Scale (HADS) was used in this study [17]. The questionnaire has a 14-item scale with two subscales: the Hospital Anxiety and Depression Scale-Anxiety (HADS-A) and the Hospital Anxiety and Depression Scale-Depression (HADS-D). This instrument includes 14 statements with four choices for each item. The odd items measure anxiety levels (HADS-A), and the even items measure depressive symptoms levels (HADS-D) [17]. The HADS was reporting subscale of HADS-A and HADS-D separately. The subscale of HADS-A was reported and published in a journal [15].

The responses of the HADS-D subscale comprise one of four choices: never (0), mild (1), moderate (2) and severe (3). To assess levels of depressive symptoms, the possible scores of HADS-D range from 0 to 21. Higher scores denote more depressive symptoms [19]. The best cut-off point of HADS-D subscale was 8. It has a sensitivity of 93.2% and a specificity of 90.8% for the HADS-D subscale. Depressive symptoms was indicated if the scores were 8 and above, while no depressive symptoms was apparent if the scores were below 8 [17].

The Malay-version Medical Outcome Study (MOS) Social Support Survey consists of 16 items and four dimensions, namely, emotional/informational (six items), tangible (three items), affectionate (three items) and positive social interaction support (four items) [18]. Items are rated on a five-point scale ranging from 1 (none of the time) to 5 (all the time), with higher scores indicating more support. The composite reliability of the domains ranged from 0.649 to 0.903, the average variance of the domains from 0.390 to 0.699 and the Cronbach’s alpha of the domains from 0.616 to 0.902. Social support was included in the analysis as an important confounder that may influence depressive symptoms levels in healthcare providers [14,15].

### Statistical analysis

The data entry and analysis were conducted using IBM SPSS Statistics version 26.0 (IBM Corp., NY). The data were checked and cleaned prior to the analysis. The distributions and frequencies were examined. All continuous variables were expressed as mean and 95% CI. Meanwhile, frequency and percentage for categorical variables. An independent *t*-test and analysis of covariance (ANCOVA) were performed [14,15].

### Ethics approval

Ethics approvals were obtained from the Universiti Sains Malaysia Human Research Ethics Committee (USM/JEPeM/COVID19-10) and the Ministry of Health Medical Research Ethics Committee (NMRR-20-703-54576). The questionnaire was anonymous, and the responses were confidential. The respondents were asked to answer the questionnaire as truthful as possible. They were to indicate their experiences and feelings, and there were no right or wrong answers.

## Results

The response rate of the study was 95.6%. A total of 306 healthcare providers participated in this study: frontline healthcare providers (n = 160) and non-frontline healthcare providers (n = 146). The majority of the healthcare providers were female, Malay, had diploma education, married, paramedics, working at the medical department and no working shift (Table 1).

**Table 1:** Socioeconomic characteristics of the frontline and non-frontline healthcare providers [14,15].

	Frontliners (n = 160)		Non-frontliners (n = 146)	
Variables	n	(%)	n	(%)
Age (years)^a^	37.96	(6.21)	38.46	(7.13)
Household income (MYR)^a^^,^^b^	5754.73	(3121.83)	4369.14	(2726.48)
Number of children^a^	2.46	(1.53)	2.27	(1.57)
Duration of employment (years)^a^	12.66	(5.84)	12.43	(7.01)
Gender:				
Male	19	(11.9)	42	(28.8)
Female	141	(88.1)	104	(71.2)
Race:				
Malay	156	(97.5)	145	(99.3)
Non-Malay	4	(2.5)	1	(0.7)
Education level:				
Diploma	134	(83.8)	135	(92.4)
Bachelor	18	(11.2)	9	(6.2)
Master	8	(5.0)	2	(1.4)
Marital status:				
Married	133	(83.1)	132	(90.4)
Unmarried	27	(16.9)	14	(9.6)
Occupation:				
Paramedic	137	(85.6)	139	(95.2)
Medical staff	23	(14.4)	7	(4.8)
Department:				
Medical	54	(33.8)	24	(16.4)
Emergency	2	(1.3)	51	(34.9)
Intensive Care Unit	67	(41.9)	0	(0.0)
Surgical	14	(8.8)	56	(38.4)
Obstetric Gynaecology	5	(3.1)	0	(0.0)
Others	18	(11.2)	15	(10.2)
Shift work:				
No	154	(96.2)	137	(93.8)
Yes	6	(3.8)	9	(6.2)
Social support:				
Emotional support^a^	70.31	(25.44)	58.48	(27.93)
Tangible support^a^	64.43	(28.21)	56.22	(29.13)
Affectionate support^a^	74.74	(25.55)	62.84	(28.70)
Positive social interaction^a^	74.18	(24.48)	62.46	(27.51)

^a^ Expressed as mean (standard deviation)

^b^ Missing values (frontline healthcare providers, n = 128; non-frontline healthcare providers, n = 87).

The HADS items for depressive symptoms (HADS-D) showed that the mean score for the non-frontline group was higher than that of the frontline group for most of the scale items. For items 4 and 12, the mean score of the frontline group was higher compared to that of the non-frontline group (Table 2).

**Table 2:** Hospital Anxiety and Depression Scale-Depression items among healthcare providers.

		Frontliners (n = 160)		Non-frontliners (n = 146)	
	Items	Mean	(SD)^a^	Mean	(SD)^a^
2	I still enjoy the things I used to enjoy	0.57	(0.86)	0.75	(0.91)
4	I can laugh and see the funny side of things	1.08	(1.14)	0.79	(1.03)
6	I feel cheerful	0.59	(0.69)	1.14	(0.97)
8	I feel as if I am slowed down	0.96	(0.67)	1.26	(0.79)
10	I have lost interest in my appearance	0.43	(0.73)	0.49	(0.75)
12	I look forward with enjoyment to things	2.57	(0.81)	2.14	(1.04)
14	I can enjoy a good book or radio or TV program	0.64	(0.92)	0.95	(1.10)

^a^ Standard deviation.

The frequency of mean HADS score for depressive symptoms (score >8) was 99 (32.4%): 44 (27.5%) for the frontline healthcare providers and 55 (37.7%) for the non-frontline healthcare providers. The overall mean (SD) HADS score of depressive symptoms for healthcare providers was 7.17 (2.98). The mean score for the frontline healthcare providers was normally distributed, ranging from 1 to 15, with a mean (SD) of 6.84 (2.98). The mean score for the non-frontline healthcare providers was also normally distributed and ranged from 1 to 15 with a mean (SD) of 7.53 (2.95). The independent *t*-test analysis showed a significant difference in depressive symptoms scores (*p* = 0.043), with non-frontline healthcare providers showing a 0.69 mean score difference compared to the frontline healthcare providers. ANCOVA showed a significant difference in the estimated marginal mean of the depressive symptoms scores between the non-frontline and frontline healthcare providers, with a mean difference of 0.75 after adjusting for gender, duration of employment and social support (*p* = 0.036. ANCOVA confirmed the higher depressive symptoms score among the non-frontline healthcare providers by a unit mean score of 7.35 (95%CI: 6.83; 7.88) after adjusting for gender, duration of employment and social support (Table 3).

**Table 3:** Comparison of depressive symptoms levels between frontline and non-frontline healthcare providers.

Groups	N	Desc mean^a^	(SD)^b^	EMM^c^ (95% CI^d^)	Mean diff^e^ (95% CI^d^)	F stat^f^ (*df*^g^)	*P* value^h^
Frontline	160	6.84	(2.98)	6.61 (6.04; 7.17)	−0.75 (−1.45; −0.05)	4.44 (1)	0.036
Non-frontline	146	7.53	(2.95)	7.35 (6.83; 7.88)			

^a^ Descriptive mean

^b^ Standard deviation

^c^ Estimated marginal mean = mean after adjusting covariates

^d^ Confidence interval

^e^ Mean difference

^f^ F statistic

^g^ Degree of freedom

^h^ Analysis of covariance. Adjusted for gender, duration of employment and social support.

## Discussion

Healthcare providers bear a high burden of responsibility, particularly in the public health response to the COVID-19 pandemic. This responsibility may have a significant impact on their physical and psychological health. Depressive symptoms is one of the common psychological impacts experienced by healthcare providers who respond to outbreaks and pandemics. In this study, the overall mean score of HADS-D indicated that healthcare providers had lower levels of depressive symptoms. However, when a comparison was made between frontline and non-frontline group, the frontline healthcare providers had lower levels of depressive symptoms compared to the non-frontline healthcare providers.

Although there was a significant difference, the levels for both groups were lower than those required to denote clinical depressive symptoms, as indicated by the mean scores for both groups, which were less than eight. This might be influenced by lower cases of COVID-19. The total reported COVID-19 cases in that state was 160 cases until the end of July 2020 [14].

A study in Singapore had a similar result: the non-medical healthcare workers had a higher mean score for depression compared to the medical healthcare workers [20]. However, this result contradicted that of a study in China, which found that the medical health workers had a higher prevalence of depression than the non-medical health workers [21]. Furthermore, there was no significant difference in depression status between the two groups in a study in Oman [22]. The study in other state of Malaysia found that most healthcare workers working in government healthcare facilities at the study location did not have depression or had normal levels of depression [9]. Most (66.7%) of them were directly involved in the management of COVID-19 cases.

The Malaysian study has also found evidence that depression is strongly intercorrelated with anxiety, stress and chronic fatigue [9]. A shortage of frontline staff may result in non-frontline staff being deployed in frontline positions [23], which may trigger anxiety, stress and depression [10]. This is because non-frontline healthcare providers have less experience, skills and training to face the pandemic compared to frontline healthcare providers who are well prepared for this work both mentally and physically and have the necessary knowledge, skills and training [24].

Staff shortages caused the management to reshuffle placements and rescheduled the working hours. Changing in the working environment with lack of skills and less experiences may affect their working performance and may cause depression if they failed to achieve the job requirement. This situation will escalate if these non-frontline healthcare providers need to work longer hours and take on a greater workload in terms of shifts and patients [25] as this may cause chronic fatigue. Burnt out, one of the impacts of this situation may lead to psychological disorders with more prolonged exposure. This finding was supported by a study in Shanghai [26]. The researchers suggested that after a considerable number of healthcare workers were deployed to Wuhan to control the initial COVID-19 outbreak, non-frontline healthcare providers experienced more fatigue due to the higher workload required compared to the daily healthcare services they generally provided for other health issues.

Tan et. al. [20] determined that non-frontline healthcare providers had higher levels of depression than frontline healthcare providers because non-frontline healthcare providers have less access to formal psychological support. This observation was similar to that of the current study since non-frontline healthcare providers have less social support than frontline healthcare providers. In a study by Geoffroy et al. [27], psychological support services were first implemented for frontline healthcare providers before being extended to non-frontline healthcare providers after administrators realized that non-frontline healthcare providers were also experiencing psychological distress. However, in a separate study, some healthcare staff refused these services, stating that they did not have any problems but only needed rest [28]. They have been in longer working hours with a workload burden [25] and in a stressful working environment.

Non-frontline healthcare providers may have less medical information regarding COVID-19 and less intensive training on personal protective equipment and infection control measures [20]. Medical information on COVID-19 from trusted sources is very important but particularly so for non-frontline healthcare providers. They need to avoid unpleasant and non-verified news disseminated via social media concerning the number of patients and the death tolls as this can make them mentally very sensitive and fragile, which may lead to depression [29]. Guidelines and policies should, therefore be put in place to help prevent this. Compared to non-frontline healthcare providers, frontline healthcare providers have more access to accurate information and may feel closer to key decision-makers [30]. Hands-on training with guided checklists, especially regarding the donning and doffing of personal protective equipment, airway management, and cardiopulmonary resuscitation, have been essential and useful in managing unfamiliar outbreaks as COVID-19 [31]. It has further been recommended that non-medical healthcare workers receive appropriate training to ensure they have an understanding of the use of infection control measures [20].

As limitations of the study, a cross-sectional design study cannot be generalized to other population. However, this study gave evidence that non-frontline healthcare providers experienced more depressive symptoms than frontline healthcare providers. Meanwhile, a self-administered questionnaire applied in this study to reduce face-to-face interviews may produce common method bias and social desirability bias. Anonymous survey was used with the hope it may reduce such biases. There were no psychometric properties of the Hospital Anxiety and Depression Scale (HADS) conducted among healthcare providers in Malaysia. Future study of the validity and reliability of the HADS among healthcare providers in Malaysia is needed since the mental health of the healthcare providers is a concern and received increased attention during the pandemic.

## Conclusion

Depressive among healthcare providers–whether from frontline or non-frontline groups–needs to be addressed to prevent some of the established negative ramifications of depressive symptom. These groups need each other in complementing health services. Depressive score was higher in non-frontline healthcare providers compared to frontline healthcare providers. Non-frontline healthcare providers were affected by more workload burden and fear of been infected with COVID-19 as the workplace was a red zone of COVID-19. Both groups must be supported psychologically and treated equally based on their conditions. Improving the healthcare system in these ways would be beneficial in maintaining the quality of healthcare services, especially during the current COVID-19 pandemic.

## Abbreviations

COVID-19: Coronavirus disease 2019
CI: Confidence interval
SD: Standard deviation
HADS: Hospital Anxiety and Depression Scale
MOS: Medical outcome study
HADS-A: Hospital Anxiety and Depression Scale for anxiety
HADS-D: Hospital Anxiety and Depression Scale for depression
ANCOVA: analysis of covariance
